# Insights From Immigrant and Refugee Communities Regarding COVID-19 Needs and Opportunities: A Mixed Methods Study

**DOI:** 10.1016/j.focus.2023.100099

**Published:** 2023-04-27

**Authors:** Nicole A. Stadnick, Kelli L. Cain, William T. Oswald, Paul L. Watson, Jesse Nodora, Shelia L. Broyles, Angel A. Lomeli, Arleth A. Escoto, Marina Ibarra, Raphael Lagoc, Borsika A. Rabin

**Affiliations:** 1 Department of Psychiatry, University of California San Diego, La Jolla, California; 2 Dissemination & Implementation Science Center (DISC), Altman Clinical and Translational Research Institute, University of California San Diego, La Jolla, California; 3 Child & Adolescent Services Research Center (CASRC), Department of Psychiatry, University of California San Diego, San Diego, California; 4 The Herbert Wertheim School of Public Health and Human Longevity Science, University of California San Diego, La Jolla, California; 5 The Global Action Research Center, San Diego, California; 6 Department of Pediatrics, University of California San Diego, La Jolla, California; 7 Department of Obstetrics, Gynecology, & Reproductive Sciences, University of California San Diego, La Jolla, California; 8 Department of Radiation Medicine and Applied Sciences, University of California San Diego, La Jolla, California; 9 Altman Clinical and Translational Research Institute, University of California San Diego, La Jolla, California; 10 Moores Cancer Center, University of California San Diego, La Jolla, California

**Keywords:** Mixed methods, immigrants, refugees, community engagement, COVID-19, trusted sources

## Abstract

**Introduction:**

This mixed-methods study describes processes to actively engage underserved, immigrant, and refugee communities in COVID-19 vaccine efforts to cocreate culturally relevant resources and dissemination.

**Methods:**

A survey on healthcare characteristics and COVID-19 attitudes was deployed between March and November 2021 followed by listening sessions conducted in December 2021. All participants were recruited through the project's Community Advisory Board. The survey, completed by 77 adults, was available in English, Spanish, Burmese, Kizigua, and Karen. Listening sessions were led by trained, multilingual, and multicultural interviewers.

**Results:**

Doctors/healthcare providers were rated as the most trusted messengers of COVID-19 information; however, trusted sources varied across communities. Data from 3 listening sessions (with a total of 14 participants) expanded survey findings with a focus on trusted sources of COVID-19 communication.

**Conclusions:**

This study confirmed the importance of healthcare professionals as trusted messengers for COVID-19 information among underserved communities. Qualitative data highlighted the importance of schools, ethnically based community organizations, and friends/family with health and English literacy skills for immigrant and refugee communities. Findings suggest opportunities for collaboration with specific trusted sources for future public health dissemination efforts.

## Highlights

• Immigrant and refugee communities reported common and unique trusted messengers.
• Healthcare providers were the most trusted messengers.
• Schools, ethnically based community organizations, and friends/family were also important.
• Collaborations with these trusted sources may aid public health dissemination.

## INTRODUCTION

There is substantial evidence that the coronavirus disease 2019 (COVID-19) pandemic disproportionately impacted and worsened the health and socioeconomic disparities experienced by communities of color in the U.S.^1^ The syndemic impacts of COVID-19 interacting with historical and pervasive social and systemic inequities for Black, Latina/o, indigenous, immigrant, and refugee communities are deservedly receiving needed attention in scientific, popular media, and policy outlets.^2^ Immigrant and refugee communities may acutely and uniquely experience social and systemic inequities that impact their health outcomes and care experiences.^3^^,^^4^

Limited health literacy (including knowledge of the U.S. healthcare system), lower English proficiency, discrimination, and experience of community violence after resettlement have been shown as indicators of poorer health outcomes and challenges in accessing and receiving healthcare services.^5^ The immigrant and refugee communities in the U.S. are from diverse geographic and cultural backgrounds and likely experienced varied immigration experiences.^6^ These factors may influence their expressions and perspectives of health and healthcare consumption. The vast diversity of immigrant and refugee communities and the complex immigration and refugee resettlement experience require cultural sensitivity and competence by U.S. hosts. This is particularly important in healthcare contexts, which are often the first point of interaction after arriving in the U.S.^7^

San Diego has been a major hub for immigrant and refugee communities.^2^ In fiscal years 2020–2021, San Diego resettled the highest number of refugees in the state of California.^8^ Approximately 27% of the city of San Diego's population is foreign-born, with individuals immigrating from at least 115 countries and territories. The most prevalent foreign-born communities in the City of San Diego are from Latin America, Asia, and Europe. Those from Africa have contributed most to the growth of foreign-born communities in San Diego over the past several years.^9^

A recent report and publication^10^ produced in collaboration with the Partnership for the Advancement of New Americans documented how the COVID-19 pandemic impacted refugee communities in San Diego. Since the start of the pandemic, refugee communities have experienced significant barriers in obtaining sustained employment, food security, safe and affordable housing, and access to health care. In addition, the complexities of anti-immigrant rhetoric have influenced perceptions of safety, leading to a decline in mental health among refugee communities. COVID-19 has exacerbated the health disparities that refugee communities face, contributing to low testing rates, lack of trust in their healthcare providers, and decreasing rates in vaccine uptake owing to vaccine hesitancy.^2^

The NIH launched several rapid response funding opportunities for community-engaged research efforts to reduce disparities in COVID-19 clinical trial participation, access to care, and vaccine uptake.^11^ One such NIH initiative is the Community Engagement Alliance Against COVID-19 Disparities (CEAL),^12^ which includes community−academic teams in 11 states throughout the U.S. and focuses on COVID-19 awareness and education research for communities most affected by the COVID-19 pandemic. The CEAL team in California involves a community−academic network of 11 academic institutions in California, including the University of California San Diego. The purpose of this mixed-methods study is to describe findings from the University of California San Diego CEAL team's project focused on multilevel barriers, facilitators, and processes to engage underserved communities in San Diego in COVID-19 vaccine efforts to inform the cocreation of culturally relevant community resources and dissemination approaches. This paper specifically focuses on the role of trusted messengers of health information during the COVID-19 pandemic across multiple underserved communities.

## METHODS

### Study Design

This mixed-methods study used an explanatory sequential design. A quantitative survey regarding health and healthcare characteristics, COVID-19 attitudes, and experiences was first deployed, followed by a series of qualitative listening sessions designed to expand upon specific survey findings. The University of California San Diego IRB approved this study. All participants provided their verbal or written consent to share their data.

### Procedures

Participants invited to complete the quantitative survey were recruited through the project's Community Advisory Board (see Stadnick et al.^13^ for more details) and Community Partner (the Global Action Research Center) by sharing a link or paper copies of the survey to their community networks. The survey was available and distributed in the following languages: English, Spanish, Burmese, Kizigua, and Karen. The goal was to receive survey responses from 15 members in each of the following communities: African American, Latina/o, Burmese, Karen, and Somali Bantu. The survey took approximately 30 minutes to complete. Survey data were collected between March and November 2021. Participants received a $20 gift card for completing the survey.

A subset of survey respondents was invited to participate in a 1-hour qualitative listening session to expand on their survey responses. Individuals were invited to participate in a listening session if they (1) agreed to be contacted about a follow-up listening session when they completed the survey and (2) identified as being a member of the Karen, Latina/o, or Somali Bantu community. These 3 community groups were selected to participate in a listening session because they are among the most prevalent immigrant and refugee communities in the local study area and because the study's Community Partner (the Global Action Research Center) has established relationships with ethnically based organizations serving these community groups. The sample goal for each listening session was 6 participants per community. A total of 3 listening sessions were conducted in each of the following languages: Karen, Spanish, and Kizigua. Listening sessions were conducted in December 2021 and January 2022 through Zoom or in person. Listening session participants received a $40 gift card.

To facilitate culturally appropriate and authentic engagement, 3 community specialists were hired and trained as research staff to support quantitative and qualitative data collection for communities who spoke Burmese, Karen, and Kizigua. Community specialists were nominated by the project's Community Advisory Board for their leadership and engagement skills within their respective communities and their fluency in multiple languages spoken by local immigrant and refugee communities. The community specialists completed the University of Illinois Chicago Human Subjects Research CIRTification Online program along with an initial 1-hour training with University of California San Diego research staff regarding the conduct of in-person and remote listening sessions and data collection procedures. The specific activities that community specialists led included the administration of the quantitative survey, the conduct of listening sessions in specific languages, and the translation/transcription of data into English. These community specialists received a stipend ($400 for survey data collection activities and $400 for listening session data collection activities) for completing these activities.

### Measures

The quantitative survey included 27 items from the CEAL Common Survey (Version 1) developed by National Needs Assessment and Evaluation Working Group. The 27 items assessed domains of (1) COVID-19 preventive behaviors and trusted sources of pandemic information, (2) COVID-19 clinical trial participation, (3) COVID-19 vaccine attitudes and experiences, (4) sociodemographic characteristics, and (5) impacts of COVID-19 on daily activities. Two additional items were developed for this project related to motivations for COVID-19 vaccination, and 3 items related to vaccine status were also developed. In this article, we prioritized examining and reporting on the domain of trusted sources of pandemic information.

The listening session guide was iteratively developed in partnership between the academic and community partners after reviewing findings from the survey data and was designed to expand upon the survey findings in service of the larger project goal to inform culturally relevant community resources and dissemination approaches. The final listening session guide focused on further understanding the trusted sources of COVID-19 information in San Diego immigrant and refugee communities and which sources should be used when communicating information about public health issues to improve reach and trust. A semistructured interview was led by 1 facilitator who posed the following questions: (1) *Where did you get your information about the COVID-19 pandemic?*, (2) *Which sources did you trust and why?*, (3) *What did you do with the information you received?*, (4) *What can be done to ensure you have easy access to trusted information?*, and (5) *Would you like to share anything else about your experience receiving information related to the COVID-19 pandemic?*

### Data Analysis

Survey data were analyzed descriptively by community group (African American, Latina/o, Karen, Burmese, Somali Bantu). The listening session qualitative data were analyzed using a rapid analytic approach^14^ that involved first developing a templated matrix of summary responses from each question posed and responded to in the listening sessions. All qualitative data were professionally transcribed and translated into English. After this, the community−academic team reviewed the matrix of qualitative summary responses and codeveloped emergent themes regarding trusted sources of COVID-19 and health information. Because the qualitative data were intended to expand on the survey findings related to the reasons why certain sources of information were trusted (or not), particular attention was focused on similarities and differences in qualitative responses across the listening sessions on the basis of the community group that participated. A joint display was produced to triangulate data from the survey and listening session data.

## RESULTS

A total of 77 adults consented to participate and shared their responses on the paper (58%) or online (42%) surveys. The approximate response rate is 65% on the basis of the number of individuals who started an online survey (*n*=49) relative to the number who completed an online survey. A subset of 14 adults from the survey sample and who expressed interest in participating in a follow-up qualitative data collection participated in a listening session. Table 1 reports the characteristics of the survey and listening session respondents.

**Table 1:** Sample Characteristics

Characteristics	Survey respondents, *n*=77	Listening session participants, *n*=14
Preferred language, *n* (%)		
Spanish	18 (23.4%)	4 (28.6%)
Karen	15 (19.5%)	4 (28.6%)
Kizigua	15 (19.5%)	6 (42.9%)
Burmese	15 (19.5%)	—
English^a^	14 (18.2%)	—
Gender, *n* (%)		
Female	63 (81.8%)	11 (78.6%)
Male	12 (15.6%)	3 (21.4%)
Other/not reported	2 (2.6%)	—
Age		
18–29 years	25 (32.5%)	4 (28.6%)
30–39 years	10 (12.9%)	2 (14.3%)
40–49 years	16 (20.8%)	3 (21.4%)
50–59 years	13 (16.9%)	4 (28.6%)
60–77 years	10 (12.9%)	1 (7.1%)
Employment status, *n* (%)^b^		
Part or full-time employed	27 (35.1%)	4 (28.6%)
Staying at home to care for the home or others	21 (27.3%)	4 (28.6%)
Not working	12 (15.6%)	2 (14.3%)
Retired	8 (10.4%)	1 (7.1%)
Attending school	4 (5.2%)	–
Other	9 (11.7%)	2 (14.3%)
None of the above/no response	10 (13.0%)	3 (21.4%)
Household size, *n* (%)		
1–2	12 (16.2%)	1 (7.1%)
3–5	43 (58.1%)	8 (57.2%)
6–8	19 (25.7%)	5 (35.7%)
Highest degree or level of school completed, *n* (%)		
Less than high school	30 (39.0%)	9 (64.3%)
Some high school	8 (10.4%)	2 (14.3%)
High school graduate or GED	15 (19.5%)	2 (14.3%)
Associate's or technical degree	6 (7.8%)	1 (7.1%)
Bachelor's degree	7 (9.1%)	—
Graduate degree	6 (7.8%)	—
Prefer not to answer	3 (3.9%)	—
Trusted sources of COVID-19 information (based on endorsed ratings of trust a great deal), *n* (%)		
Doctor/healthcare provider	55 (71.4%)	8 (57.1%)
U.S. Coronavirus Task Force	51 (66.2%)	7 (50.0%)
U.S. government	40 (51.9%)	6 (42.9%)
Faith leader	30 (39.0%)	6 (42.9%)
Closer friends/family members	30 (39.0%)	3 (21.4%)
Work peers/classmates/peers	17 (22.1%)	2 (14.3%)
News on radio, TV, online, newspapers	17 (22.1%)	3 (21.4%)
Social media contacts	11 (14.3%)	1 (7.1%)

a Identified as Black or African American.

b Respondents could select more than 1 option.

From the survey data, results revealed that the most trusted sources for accurate information about COVID-19 reported by all community groups were doctors/healthcare providers, the U.S. Coronavirus Task Force, the U.S. Government, and closer friends or family. The least trusted sources were social media and news on the radio, on the TV, online, or in the newspaper. Faith leaders were the most trusted source among Somali Bantu participants and the least trusted source among English-speaking, African American participants.

**Figure 1:**
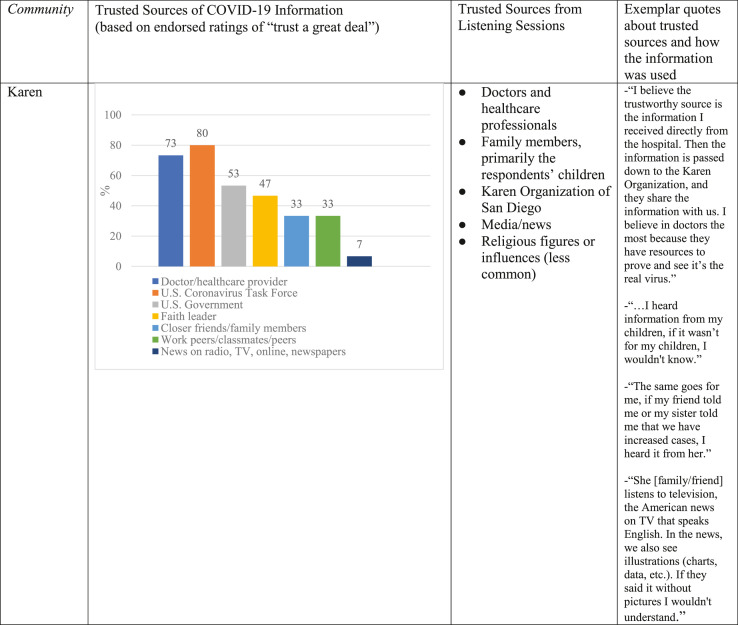

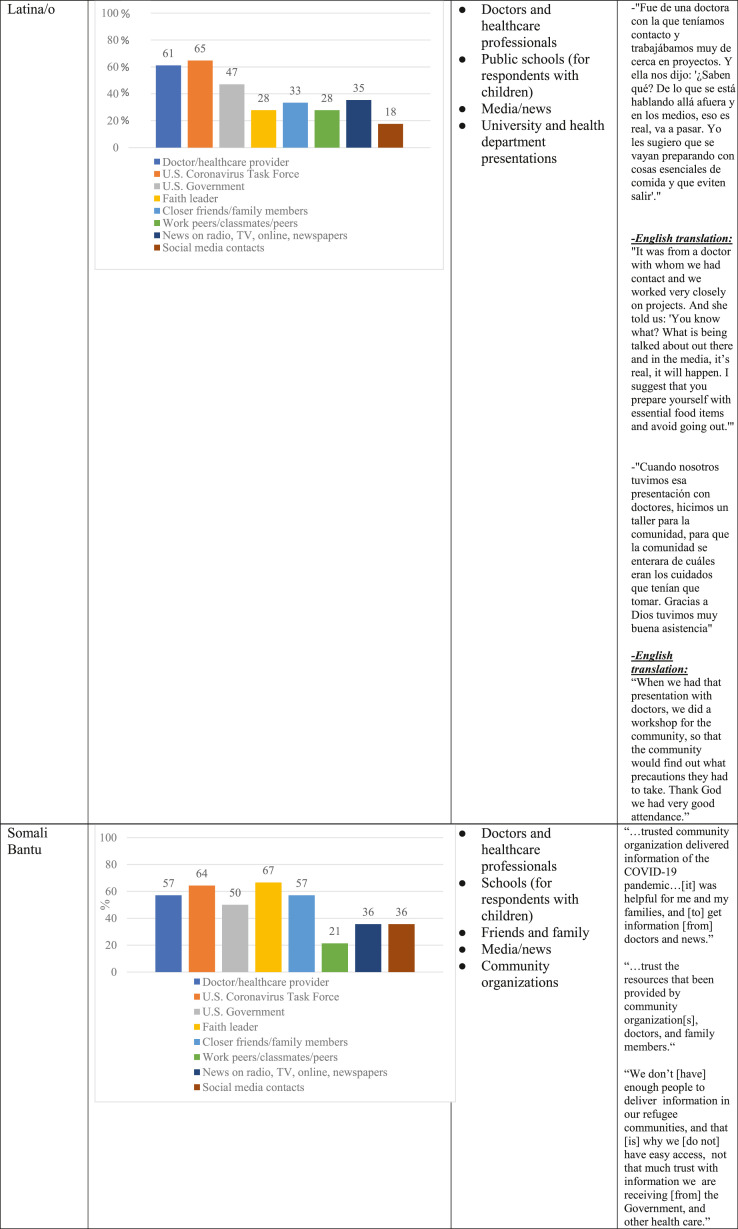
Joint display reporting expanded insights about COVID-19 trusted information sources.

Qualitative data from 3 listening sessions conducted in Karen, Spanish, or Kizigua expanded survey findings by enriching the understanding of the breadth of trusted sources of COVID-19 communication and reasons why specific sources were trusted by unique communities. In addition to healthcare providers, the Karen, Latina/o, and Somali Bantu communities described the important and unique roles of their family, children, and friends in receiving up-to-date information and recommendations for protecting their health. For those with school-aged children, listening session participants reported the usefulness of receiving communication from their children's school about COVID-19 preparedness and response. Karen and Somali Bantu participants uniquely shared the essential role of local, ethnically based community organizations that disseminated COVID-19 health information and resources in their preferred language and easily accessible communication modes. Figure 1 is a joint display that juxtaposes survey data about rank-ordered trusted COVID-19 messengers with qualitative listening session data with respect to the rationale for trusted messengers and the utility of COVID-19 information accessed.

## DISCUSSION

This mixed-methods CEAL study offers important and understudied perspectives from underserved, immigrant, and refugee communities in Southern California about trusted messengers of COVID-19 information. Overall, findings confirmed the key role of healthcare professionals as trusted disseminators of COVID-19 public health information and guidance. An additional expansion of this information with qualitative data highlighted the importance of schools, ethnically based community organizations, and friends or family with health and English literacy skills for immigrant and refugee communities to access, understand, and act upon COVID-19 prevention and response recommendations. These findings are generally consistent with results from recent publications that examined differences in trusted messengers for subgroups of underserved communities.^15^ A recent national report^16^ identified doctors as one of the key messengers of information owing to their honesty, consistency, and lack of bias in conveying important information. When exploring cultural and ethnoracial differences in preferred health information sources on the basis of data from the Health Information National Trends Survey, Weldon^17^ and colleagues found that doctors were ranked as a primary source of health information followed by government, family and friends, and charity and faith-based organizations. Furthermore, Weldon et al.^17^ reported that trust in faith-based organizations was associated with limited English proficiency. Findings from this study indicated that the role of faith-based leaders as trusted messengers of health information varied across cultural groups whose primary languages were not English. Specifically, the Somali Bantu community endorsed higher ratings of trust in faith leaders than Latino/a and Karen community members.

### Limitations

This study has several strengths and limitations. A noteworthy strength is the successful engagement of immigrant and refugee communities living in San Diego, one of the primary resettlement hubs in the U.S., in COVID research and service. To invite participation from these communities, the CEAL team relied on the expertise of Community Advisory Board members and community specialists who identified as members of local immigrant and refugee communities and had strong relationships with ethically based community organizations. Following the principles for community engagement,^18^ both the Community Advisory Board members and community specialists were provided with compensation for their time and expertise to meaningfully engage their community networks. This study is also strengthened by the mixed-methods design that allowed the quantitative survey data to be contextualized and further detailed and described by the qualitative listening session data, and finally, all data collection and interpretation were conducted by trained investigators using the language from the respondent's country of origin.

Balanced with these strengths are limitations in the design and potential generalizability of findings. The primary limitation is the small sample sizes within each of our diverse community groups. The efforts to engage these underserved, immigrant, and refugee communities in culturally competent and meaningful ways necessitated dedicated financial, personnel, and time resources that precluded a larger sample size for the survey or listening sessions. For example, the Somali Bantu community engaged in our study primarily uses oral language to communicate; therefore, our community specialist needed to complete the survey in an interview style for all participants. Because of the relatively small sample size for the quantitative and qualitative data and because we did not have the opportunity to conduct a listening session with English-speaking participants, caution is recommended in generalizing findings to other underserved, immigrant, and refugee communities and those outside of Southern California.

Another limitation is that we were not able to report on a larger number of demographic characteristics. Although we included preferred language, age, gender, employment status, education, and household size, we aimed to minimize the response burden in our survey and listening sessions. As such, we identified, with our community partner, the minimal number of important demographic data to collect to maximize our opportunity to ask more substantive questions about trusted sources of health information.

## CONCLUSIONS

Taken together, our findings suggest several opportunities to actively engage specific trusted sources in future public health dissemination efforts. Healthcare providers are clearly central to trusted health dissemination for the immigrant and refugee communities in our study. To expand healthcare provider influence, it is recommended that health systems develop and nurture strategic partnerships with local elementary and high schools, ethnically based organizations, and community-based organizations for public health preparedness and response initiatives. Community health workers may be particularly helpful to reinforce and clarify health guidance initially relayed by healthcare professionals. Associated resources to support these partnerships are essential, including access to up-to-date public health surveillance, best practices, community resources, and staff who can translate this public health guidance into culturally and linguistically relevant materials.

Understanding the priorities and preferences of underserved communities, a priori, is especially critical to address the health equity gap that exists for these groups. Specifically, providing information about critical public health concerns such as the COVID-19 pandemic using messengers who are most trusted by communities of color and immigrant communities has the potential to increase their acceptance and uptake of this information and leads to better public health outcomes.

## Appendix Supplementary materials

Image, application 1
