# Supplementary material for: Insights From Immigrant and Refugee Communities Regarding COVID-19 Needs and Opportunities: A Mixed Methods Study
Source: AJPM Focus. 2023 Apr 27;2(3):100099. doi: 10.1016/j.focus.2023.100099 (PMC10133022; doi:10.1016/j.focus.2023.100099)
Supplement: Supplementary file 1 [file mmc1.docx]

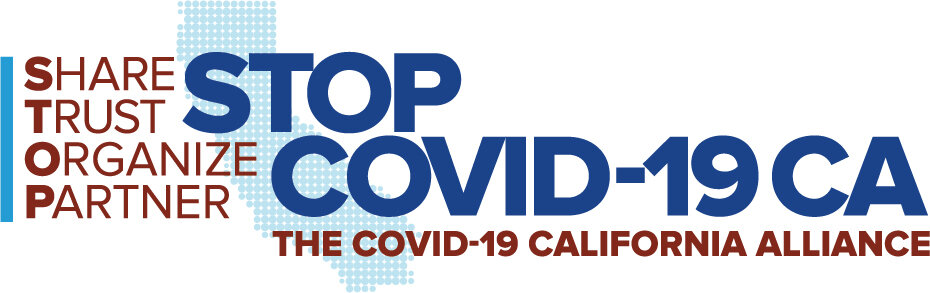


**COMMUNITY SURVEY**

Thank you for taking the time to complete this 30 minute survey. We are interested in understanding your attitudes and beliefs related to participation in COVID-19 research and accessing COVID-19 care. We will also ask some questions about you and your household so we can better understand your needs. At the completion of this survey, you will receive a $20 gift card.

**Let’s get started**

**Q01. What is today’s date** _______________________________

**Q02. What are the last 4 digits of your cell phone number?** We are asking to ensure we only get one survey per person.

______________________

# COVID‑19 Prevention

**Coronavirus Disease 2019 (COVID-19) is a disease caused by the new coronavirus. The first set of questions asks what you think about COVID‑19 and how to stay safe from COVID‑19.**

1. In the past 7 days, how often have you chosen to do each of the following when in public to keep yourself and others safe from COVID‑19? *Do not include things you were required to do, such as wear a mask while visiting a store. (Select one response for each row.)*

|  | All of the time | Very often | Some of the time | Never |
| --- | --- | --- | --- | --- |
| Wore a face covering or mask |  |  |  |  |
| Washed my hands with soap or used hand sanitizer several times per day |  |  |  |  |
| Stayed at least 6 feet away from other people who are not from my household |  |  |  |  |

1. Have you ever been tested for COVID‑19?

- Yes
- No

1. How much do you trust each of these sources to provide correct information about COVID‑19? *(Select one response for each row.)*

|  | Not at all | A little | A great deal | Don’t Know |
| --- | --- | --- | --- | --- |
| Your doctor or health care provider |  |  |  |  |
| Your faith leader |  |  |  |  |
| Your close friends and members of your family |  |  |  |  |
| People you go to work or class with or other people you know |  |  |  |  |
| News on the radio, TV, online, or in newspapers |  |  |  |  |
| Your contacts on social media |  |  |  |  |
| The U.S. government |  |  |  |  |
| The U.S. Coronavirus Task Force |  |  |  |  |
| Other (please write in): |  |  |  |  |

COVID-19 Clinical Trials

Now we are going to ask you some questions about COVID‑19 clinical trials.

A **clinical trial** is a kind of research study. Clinical trials study if treatments or vaccines are safe for people and if they work like they are supposed to.

Right now, clinical trials are being done across the U.S. to see if new treatments and vaccines for COVID-19 work to keep people healthy.

1. Have you ever signed up for a COVID‑19 clinical trial?

- Yes, I have signed up for a clinical trial for a COVID‑19 vaccine. **[IF YES, SKIP TO Q9]**
- Yes, I have signed up for a clinical trial for a COVID‑19 treatment. **[IF YES, SKIP TO Q7]**
- Yes, I have signed up for both COVID19 vaccine and COVID-19 treatment. **[IF YES, SKIP TO Q9]**
- No, I have never signed up for a COVID‑19 clinical trial.
- I am not sure

1. If you have never signed up for a clinical trial, are you aware of COVID‑19 clinical trials that are being done?

- Yes, clinical trials for COVID-19 vaccines
- Yes, clinical trials for COVID-19 treatments
- Yes, I am aware of clinical trials for COVID-19 vaccines and treatments
- No **[IF NO, SKIP TO Q7]**
- Not sure **[IF NOT SURE, SKIP TO Q7]**

1. Do you know what to do to sign up for a COVID‑19 clinical trial in your area?

- Yes
- No
- Not sure

1. How *willing* are you to sign up for a clinical trial for a COVID-19 vaccine?

| Not at all willing |  |  |  |  |  | Very willing |
| --- | --- | --- | --- | --- | --- | --- |
| 1 | 2 | 3 | 4 | 5 | 6 | 7 |

1. How *likely* are you to sign up for a clinical trial for a COVID-19 vaccine?

| Not at all likely |  |  |  |  |  | Very Likely |
| --- | --- | --- | --- | --- | --- | --- |
| 1 | 2 | 3 | 4 | 5 | 6 | 7 |

1. Below are sources of information of COVID-19 clinical trials. How much do you trust each of these sources to give correct information? *(Select one response for each row.)*

|  | A great deal | A fair amount | Not very much | None at all | No opinion |
| --- | --- | --- | --- | --- | --- |
| The U.S. government |  |  |  |  |  |
| Your doctor or health care provider |  |  |  |  |  |
| Your local health care clinic or hospital |  |  |  |  |  |
|  | A great deal | A fair amount | Not very much | None at all | No opinion |
| University hospitals |  |  |  |  |  |
| Companies that make drugs for medical use |  |  |  |  |  |
| People who do research |  |  |  |  |  |

# COVID-19 Vaccine

**The next questions ask about a COVID-19 vaccine. A vaccine is a substance that helps protect against certain diseases.**

Q10. Have you received the COVID-19 vaccine?

- Yes
- No **[IF NO, SKIP TO Q15]**

**ONLY ANSWER THE NEXT 4 QUESTIONS IF YOU ANSWERED YES TO Q10 (you have received the COVID-19 vaccine). IF YOU ANSWERED NO TO Q10, SKIP TO Q15.**

Q11. How many doses of the COVID-19 vaccine have you received?

- 1 dose of the Pfizer vaccine only
- 2 doses of the Pfizer vaccine
- 1 dose of the Moderna vaccine only
- 2 doses of the Moderna vaccine
- 1 dose of the Johnson and Johnson

**Q12.** Why did you decide to get the COVID-19 vaccine? (*Check all that apply*)

- I wanted to keep my family safe
- I wanted to keep my community safe
- I wanted to keep myself safe
- I have a chronic health problem, like asthma or diabetes
- My doctor told me to get a COVID-19 vaccine
- I didn’t want to get really sick from COVID-19
- I wanted to feel safe around other people
- I believed life wouldn’t go back to normal until most people got a COVID-19 vaccine
- Other (please write in):

**Q13.** What made getting your COVID-19 vaccine difficult? (*Check all that apply*)

- - There was nothing that made getting my vaccine difficult
  - It was difficult to get an appointment
  - I didn’t have transportation to or from a vaccination location
  - I didn’t know where to go for my vaccination
  - I didn’t have someone to watch my children/other people in my care while I went
  - I couldn’t take time off of work for my vaccination
  - I didn’t know how to get an appointment for my vaccination
  - I didn’t have reliable technology to book my vaccination appointment
  - Vaccination locations were too far or hard to get to
  - They didn’t speak my language at the vaccination location
  - Other (please write in):

Q14. Was there anything that made you worried when getting the vaccine? (*Check all that apply*)

- - There was nothing I was worried about when getting my vaccine
  - I’m allergic to vaccines
  - I don’t like needles
  - I was concerned about side effects from the vaccine
  - I didn’t know enough about how well a COVID-19 vaccine works
  - I didn’t trust that the vaccine would be safe
  - I didn’t believe the COVID-19 pandemic was as bad as some people say it is
  - I was worried about paying for it
  - It conflicts with my religious beliefs
  - I was worried about being asked to show documentation at a vaccine appointment
  - I was worried about catching COVID-19 by going to a vaccination location
  - Other (please write in):

ONLY ANSWER THE NEXT 2 QUESTIONS IF YOU ANSWERED NO TO Q10 (you have NOT received the COVID-19 vaccine). IF YOU ANSWERED YES TO Q10 (you have received the vaccine), SKIP TO Q17)

Q15. How likely are you to get the COVID-19 vaccine in the next 3 months?

| Not at all likely |  |  |  |  |  | Very Likely |
| --- | --- | --- | --- | --- | --- | --- |
| 1 | 2 | 3 | 4 | 5 | 6 | 7 |

**Q16.** What are some reasons you have not gotten the COVID-19 vaccine? *(Check all that apply)*

- - I’m allergic to vaccines
  - I’m not concerned about getting really sick from COVID-19
  - I don’t like needles
  - I’m concerned about side effects from the vaccine
  - I don’t think vaccines work very well
  - I don’t trust that the vaccine will be safe
  - I don’t believe the COVID-19 pandemic is as bad as some people say it is
  - I don’t want to pay for it
  - I don’t know enough about how well a COVID-19 vaccine works
  - It conflicts with my religious beliefs
  - It is difficult to get an appointment
  - I don’t have transportation to or from a vaccination location
  - I don’t know where to go for a vaccination
  - I don’t have someone to watch my children/other people in my care while I go
  - I can’t take time off of work for a vaccination
  - I don’t know how to get an appointment for a vaccination
  - I don’t have reliable technology to book a vaccination appointment
  - I am worried about being asked to show documentation at a vaccine appointment
  - I am worried about being infected with COVID-19 by going to a vaccination location
  - They didn’t speak my language at the vaccination location
  - Other (write in):­­­­­­­­­­­­­­­­ ­­­­

# About You and Your Household

**The next set of questions asks about you and your household.**

1. When was the last time you saw a doctor or other health care professional for a physical or regular check-up? *Do not include visits when you were sick.*

- Never
- Within the past 12 months/1 year
- 1 to 2 years ago
- 3 to 4 years ago
- 5 to 9 years ago
- 10 years ago or more

1. Is there a place that you usually go when you are sick?

- Yes
- No **[SKIP TO Q20]**
- Don’t Know **[SKIP TO Q20]**

1. What kind of place do you go most often for medical care? *(Select one answer)*

- Clinic or health center
- Family doctor
- Hospital ER
- Urgent care clinic at a hospital
- Urgent care clinic not at a hospital
- Retail Center (for example, in a drug store)
- Some other place: Please write in here: ____________________________________
- There is no one place I go to most often for medical care

1. Do you have any kind of health insurance or health care plan? This includes health insurance you get from your job or school, that you buy yourself, and programs like Medicare and Medi-Cal.

- Yes
- No (*If No,* did you lose health care coverage because of the COVID-19 pandemic?)
  - - Yes **[SKIP TO Q22]**
    - No **[SKIP TO Q22]**
- Don’t Know

1. What is the primary kind of health insurance or health care plan that you have now?

*(Select one answer)*

- Private health insurance through a job or school
- Insurance bought through a government exchange such as healthcare.gov
- Insurance bought from a health plan or company
- Medicare
- Medi-Gap
- Medicaid/Medi-Cal
- CHIP or kid’s state insurance
- Military health care
- Indian Health Service
- Other (please write in): ________________________________
- Don’t Know

**Q22**. The COVID-19 pandemic may cause challenges for some people, whether they get COVID-19 or not. In the past month have you or your family experienced any of the below challenges?

*(Select one response for each row.)*

|  | No, this is not a challenge | Yes, this is a minor challenge | Yes, this is a major challenge |
| --- | --- | --- | --- |
| Getting the health care I need (including for mental health) |  |  |  |
| Having a place to live |  |  |  |
| Getting enough food to eat |  |  |  |
| Having clean water to drink |  |  |  |
| Getting the medicine I need |  |  |  |
| Getting to where I need to go |  |  |  |
| Other (please write in): |  |  |  |
| Other (please write in): |  |  |  |
| Other (please write in): |  |  |  |

1. Do you speak a language other than English at home?

- Yes

If *yes*, what language(s)? (*Check all that apply)*

- - Spanish
  - Swahili
  - Arabic
  - Karen
  - Burmese
  - Somali
  - Tagalog
  - Other: _____________________
  - No
  - Prefer not to answer

1. How often do you need someone to help you read written information from your doctor or drug store?

- Never
- Rarely
- Sometimes
- Often
- Always

1. What month and year were you born? Month: _______________ Year: _____________
2. What is your gender?

- Man
- Woman
- Transgender female or trans woman
- Transgender male or trans man
- Nonbinary, genderqueer, or genderfluid
- I would describe my gender as: _________________________
- Prefer not to answer

1. Which of the following best describes how you think of yourself?

- Gay
- Lesbian
- Straight (that is, not gay or lesbian)
- Bisexual
- Other
- Prefer not to answer

1. Are you Hispanic or Latino?

- No
- Yes
- Prefer not to answer

1. Which of the following best describes your race? *(Check all that apply)*

- White
- Black or African-American
- Asian
- American Indian or Alaska Native
- Native Hawaiian or Other Pacific Islander
- Prefer not to answer

1. What is the highest degree or level of school you have completed?

- Less than high school
- Some high school
- High school graduate or GED
- Associate’s or technical degree (for example, AA or AS)
- Bachelor’s degree (for example BA, BS, or AB)
- Graduate degree (for example MA, PhD)
- Prefer not to answer

1. In 2019, what was your total household income before taxes?

- Less than $15,000
- $15,000 – $19,999
- $20,000 – $24,999
- $25,000 – $34,999
- $35,000 – $49,999
- $50,000 – $74,999
- $75,000 – $99,999
- $100,000 and above
- Prefer not to answer

1. How many people live or stay in your household right now? Include yourself, any other adults, and any children. ________ people
2. Which of the below describes your situation right now? *(Check all that apply)*

- Working for pay—part time (less than 40 hours a week)
- Working for pay—full time (40 hours a week or more)
- Working without pay (for example, as an intern)
- On leave from work
- Unemployed and looking for a job
- Unemployed and NOT looking for a job
- Retired from work
- Staying at home, taking care of the home or of others
- Not able to work because of a disability
- Going to school
- Other. Please describe: __________________________________________________
- Prefer not to answer

**Q34.** We would like to invite you to participate in a ***60-minute Listening Session*** to expand on your responses. If interested, please enter your name, e-mail address and phone number. You will be offered $40 for your time. We will schedule a time that is convenient for you.

***As a reminder, your responses to this questionnaire as well as any comments made in the listening session will only be shared among the research team and will never be associated with your name.***

Name: _________________________________________

Email: _________________________________________

Phone: _________________________________________

**Thank you for completing this survey!**

Please provide your mailing address, email and phone number for us to send you the

**$20 gift card** **for your survey completion.**

Name: __________________________________________________________

Mailing address: ___________________________________________________

Email: ____________________________________________________________

Phone: ___________________________________________________________
